# Transactions of the Philadelphia College of Physicians

**Published:** 1848-01

**Authors:** 


TRANSACTIONS OF THE PHILADELPHIA COLLEGE OF PHYSICIANS.

The Summary af the Transactions of the College of Physicians of Philadelphia, from June to November, 1847, has been published, and contains its usual variety of practical matter. The subject of *ship fever* is discussed at considerable length. The disease as it appeared in Philadelphia demanded invariably a stimulant and tonic treatment. When patients could take nutriment and stimuli freely, they generally rallied in a few days, though they regained strength very slowly.

Thg Report on the Progress of Surgery, by Dr. Parish, is highly interesting. We copy the following details respecting the use of ether in surgical operations:

"The warm approval of the leading Surgeons of Boston being thus secured, the fame of etherization rapidly spread throughout New England, and the discovery was hailed in that region with enthusiastic admiration, both on account of its intrinsic importance and of the source from which it emanated. Local pride may have participated somewhat in the feelings which animated its early advocates, and a knowledge of this may, on the other hand, have induced a feeling of distrust in the cautious and timid at a distance, lest the reports of its extraordinary properties were exaggerated. Be this as it may, there does not seem to have been the same amount of interest in the discovery throughout the United States as might have been anticipated from its vast importance.

"The medical journals of the Union, although they have generally published the statements issuing from Boston, have contained, until very recently but few original communications on the subject. The profession appear to have been incredulous, or fearful of an agent for which such remarkable properties were claimed.

"As it relates to the practice of the large hospitals of the country, (with the exception stated) we have seen no official statements of the ether having been employed, and we are inclined to believe that its use has been limited. We have understood however that it has recently been employed, with satisfactory results, at the New York hospital, and we believe that there are not a few of the surgeons of that city, who are its advocates. At the Pennsylvania Hospital, in this city, it has not been tried at all, being considered by the judicious surgeons of that institution as a remedy of doubtful safety, or at least, as not sufficiently established to warrant them in its employment.

"In the city of Baltimore, as we were recently informed by an intelligent surgeon residing there, etherial inhalation was attempted in several instances during the present autumn, but without that marked benefit which would induce the experimenters to continue it—in one instance a degree of excitement was induced by it, on the eve of an amputation, which rendered it necessary to postpone the operation—and which discouraged, for the time at least, further trials.

"In regard to the practice of Philadelphia, we know of no surgical operations performed under the influence of ether, prior to the fifth month (May) of the present year. In the early part of this month, Dr. Gibson performed an amputation of the finger of a medical student who was etherized, and the result was perfectly satisfactory. In the same month, Dr. Horner extracted a cancerous breast under the use of ether, but the patient was so excited as to embarrass the operation, and the trial was considered a failure. In the summer, Dr. Pancoast used ether in an operation for hemorrhoids, with the happiest effect. Subsequently to this, Dr. J. K. Mitchell, who had visited Boston, mainly with a view of informing himself upon the subject, employed it successfully in several important cases; and within the past month, several large operations involving risk to life have been performed by Dr. Mutter upon etherized patients, at the clinic of the Jefferson Medical College. Two of these, one a case of extirpation of schirrous testicle, and the other of a fungus tumor of the eye-ball, requiring the excision of the organ, were witnessed by your committee, and the results so far as the safety and efficacy of the ether were concerned, were entirely satisfactory; since then your reporter has excised a small tumor from the head of a lady under the influence of ether; the patient being entirely conscious of the operation, and yet suffering no pain. All these patients recovered, without an unpleasant symptom, in the usual period.

"Still more recently, several important operations upon etherized patients have been performed at the clinic of the University of Pennsylvania, and the results, as we are informed, have been entirely satisfactory in preventing pain, though one patient who was subjected to an amputation of the leg, was apparently laboring under great uneasiness during the operation, notwithstanding he stated afterwards that he had not suffered. In another case of the fissure of the anus, a most painful operation, the patient was not aware that the operation had been performed until convinced of the fact by the bystanders. A case of extirpation of the eye-ball was equally successful."

